# Niobium Uptake and Release by Bacterial Ferric Ion Binding Protein

**DOI:** 10.1155/2010/307578

**Published:** 2010-04-28

**Authors:** Yanbo Shi, Ian Harvey, Dominic Campopiano, Peter J. Sadler

**Affiliations:** ^1^School of Chemistry, University of Edinburgh, King's Buildings, West Mains Road, Edinburgh EH9 3JJ, UK; ^2^CLRC Daresbury Laboratory, Warrington WA4 4AD, UK; ^3^Department of Chemistry, University of Warwick, Coventry CV4 7AL, UK

## Abstract

Ferric ion binding proteins (Fbps) transport Fe^III^ across the periplasm and are vital for the virulence of many Gram negative bacteria. Iron(III) is tightly bound in a hinged binding cleft with octahedral coordination geometry involving binding to protein side chains (including tyrosinate residues) together with a synergistic anion such as phosphate. Niobium compounds are of interest for their potential biological activity, which has been little explored. We have studied the binding of cyclopentadienyl and nitrilotriacetato Nb^V^ complexes to the Fbp from *Neisseria gonorrhoeae* by UV-vis spectroscopy, chromatography, ICP-OES, mass spectrometry, and Nb K-edge X-ray absorption spectroscopy. These data suggest that Nb^V^ binds strongly to Fbp and that a dinuclear Nb^V^ centre can be readily accommodated in the interdomain binding cleft. The possibility of designing niobium-based antibiotics which block iron uptake by pathogenic bacteria is discussed.

## 1. Introduction

**Figure 1:**
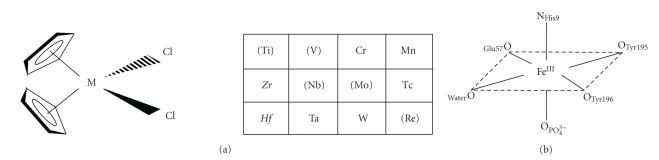
(a) Metallocene dihalides with antitumor activity. Key: (M) = maximum activity; M = sporadic activity; *M* = no activity. (b) Fe^III^ binding sites in bacterial ferric ion-binding proteins (*Neisseria gonorrhoeae or Haemophilus influenzae*).

Following the therapeutic success of cisplatin, a large number of complexes of other metals have been studied. Nonplatinum complexes are of particular interest since they may display a lack of cross-resistance with cisplatin, bringing significant benefits for chemotherapy. Metallocene dihalides and pseudohalides of general formula [Cp_2_MX_2_] (M = Ti, V, Nb, Mo; X = F, Cl, Br, I, CN, SCN; Figure 1(a)), have attracted significant interest since they have shown activity towards a wide variety of murine and human tumors [1–9]. Titanocene dichloride [Cp_2_TiCl_2_] was the first non-platinum metal complex to enter clinical trials but was eventually abandoned owing to its high reactivity in aqueous solution which gives rise to formulation difficulties [10, 11].

 Vanadocene-, molybdenocene-, and niobocene dichlorides also exhibit good activities. Niobocene dichloride (Cp_2_NbCl_2_) is an extremely potent cancerostatic agent against the Ehrich ascites tumour in CFI mice [12, 13]. Oxidation to Nb^V^ reduces the tumor inhibiting properties [14] but potentially could also reduce toxic effects. Hence there is interest in further investigation of the biological chemistry of niobium complexes [15].

 Iron is the single most important micronutrient for bacterial survival; it plays important roles in both pathogen virulence and host antimicrobial resistance [16–18]. Numerous pathogenic bacteria such as *Neisseria gonorrhoeae *and *Haemophilus influenzae *have evolved a specific protein-dependent iron-uptake system which can obtain iron from the host transferrin (Tf) and lactoferrin (Lf). The three-component system is a member of the ABC-transporter super-family (FbpABC) and critical for iron uptake is a ferric ion-binding protein (FbpA, referred to here as Fbp, a single-chain 34 kDa protein) which shuttles Fe^III^ across the periplasmic space, transporting Fe^III^ from the outer membrane to the cytoplasmic membrane [19]. This essentiality for virulence makes Fbp an ideal drug target and provides a basis for the design of novel metal-based antibiotics which combat resistance to widely used organic antibiotics.

 Structural analysis of the Fe^III^ binding site in Fbp from various bacteria has shown several different classes (Figure 1(b) shows the best characterised). All have a pair of highly conserved Tyr residues in the active site and the other metal binding residues are composed of amino acid side chains (glutamate and histidine) and anions (e.g., phosphate). These tyrosines are critical for strong metal ion binding, as confirmed by site-directed mutagenesis and crystal structures of reconstituted Fbp protein complexes with Fe^III^. These bacterial Fbps display an extremely high affinity for ferric iron (*K*_*D*_ ~ 1 × 10^−20^ M) but they also have the capacity to bind other metals. We discovered that *N. gonorrhoeae* Fbp could bind Ti, Zr^IV^, or Hf^IV^ in the metal binding cleft [20–22]. Moreover, besides binding a single metal ion we observed various metal clusters bound in an adaptable active site which appears to be able to accommodate a wide range of metals ions and anions. To further probe the specificity of this site we report for the first time studies of the binding of Nb^V^ complexes to Fbp, using a wide variety of techniques including UV-visible spectroscopy, inductively coupled plasma atomic emission spectroscopy (ICP-AES), electrospray mass spectrometry, and EXAFS. These studies are important not only for exploring a potential mechanism for niobium transport but also as a basis for the possible design of novel metalloantibiotics.

## 2. Experimental/Materials and Methods

### 2.1. Materials

[Cp_2_NbCl_2_] (Arcos), monosodium citrate (Aldrich), nitrilotriacetic acid (H_3_NTA, 99%, Aldrich, N840-7), NaH_2_PO_4_ and Na_2_HPO_4_ (BDH), Hepes (Aldrich), Tris (Aldrich), and cetyltrimethylammonium bromide (CTAB, Aldrich) were used as received. Atomic absorption standard solutions of Fe (Aldrich, cat: 30595-2), Nb (1000 ppm, niobium(V) chloride in 4% hydrofluo, VWR international Ltd. Cat: 1026410100), and P (Aldrich, cat: 20735-7) were used as supplied.

 All other chemicals were reagent grade and used as provided.

 Stock solutions of [Fe^III^(NTA)_2_]^3−^ and [Nb^V^(NTA)_n_] were prepared from iron and niobium atomic absorption standard solutions and stoichiometric amounts of H_3_NTA. The pH values of the solutions were raised slowly to *∼*5.6 and 5.26, respectively, with microliter amounts of NaOH (1 M). 

 The [Cp_2_Nb(OH)Cl_2_] stock solution was freshly prepared by sonication of [Cp_2_NbCl_2_] (2.9 mg, 0.099 mmol) in D_2_O (0.5 mL) until no solid remained (typically 0.5–1 h). As [Cp_2_NbCl_2_] is insoluble in water, oxygen was required [22] to effect oxidation to the water-soluble niobium(V) complex [Cp_2_Nb(OH)Cl_2_] [14]. Finally a yellow solution of [Cp_2_Nb(OH)Cl_2_] was obtained. This solution may also contain other hydrolysed Cp_2_Nb^V^ species as well as small amounts of hydrolysed Nb^IV^ species such as [Cp_2_NbCl(H_2_O)]^+^.

 Electrophoresis was carried out using a Bio-Rad ProteinII Minigel system (protein) and Invitrogen H5 system (DNA). GE Healthcare AKTA equipment and columns were used for chromatographic separations of proteins. Precast SDS-PAGE gels (10% bis-Tris) were purchased from Invitrogen and used according to the manufacturer's instructions.

### 2.2. Overexpression and Purification of Fbp

Fbp was overexpressed in *E. coli* TOP10 One Shot or DH5*α* cells (Invitrogen) transformed with the plasmid pTrc99A/Fbp/Ng. A single colony from freshly transformed cells was used to inoculate 5 mL of 2YT broth which contained 100 *μ*g/mL ampicillin in a sterile 10 mL vial. This culture was shaken overnight at 310 K and used to inoculate 3 liters of 2YT broth with 100 *μ*g/mL ampicillin in sterile 500 mL flasks. After the flasks were shaken overnight at 310 K, a pink cell pellet was harvested by centrifugation at 10,000 × g for 15 minutes at 277 K and stored at 253 K until use.

 Fbp was purified by a modification of the method reported previously [20, 22, 23]. The pink pellet (*∼*15 g) was defrosted at room temperature and resuspended in 150 mL of 50 mM Tris (pH 8.0) containing 2% cetyl trimethylammonium bromide (CTAB), sonicated for 5 minutes (30 second on, 30 s off), followed by stirring slowly overnight at 310 K. The white insoluble material was removed by centrifugation at 10,000 × g for 15 minutes at 277 K. The supernatant (cell-free extract) was dialysed against 5000 mL 10 mM Tris (pH  8.0) at room temperature overnight, followed by the dialysis for another 3 hours to remove CTAB and then filtered using Whatman paper (0.2 *μ*m, Fisher). The cell-free extract was applied to a RESOURCE S strong cation exchange column (6 mL, Amersham Biosciences); the column was equilibrated with 10 mM Tris buffer (pH 8.0). Unbound proteins were removed by extensive washing with low salt buffer. The target protein (Fbp) was then eluted with a linear NaCl gradient of low-to-high salt (0-1 M NaCl) over 20 column volumes in 10 mM Tris buffer. Pink fractions were collected and were analyzed by SDS-PAGE. Fbp was desalted by dialysis and concentrated by ultrafiltration (10 kDa cut-off, Amicon concentrator). The concentration of purified iron-bound, holo-Fbp protein was determined by UV absorption using *ε*_481_ = 2,430 M^−1^ cm^−1^, or *ε*_280_ = 48,900 M^−1^ cm^−1^ [24].

### 2.3. Preparation of Apo-Fbp

Iron-free, apo-Fbp was prepared by treatment of holo-Fbp solutions with 250 mM sodium citrate (pH 4.5) at room temperature for 5 hours, followed by elution with 250 mM sodium citrate (pH 4.5) on a PD-10 column (GE Healthcare), so as to give negligible absorbance at 481 nm. The apo-Fbp was then washed 6 times with 0.1 M KCl in a Centricon YM-30 microconcentrator (Amicon), and the stock solution was stored at 4°C before use. apo-Fbp concentration was determined with *ε*_280_ = 44,270 M^−1^ cm^−1^ [24].

### 2.4. UV-Visible Spectroscopy

All UV experiments were performed with 1 cm cuvettes on a computer-controlled Cary 300 spectrometer with temperature control at 298 or 310 K. For kinetic experiments, the time courses for the reactions of apo-Fbp (10 *μ*M) with 2 mol equivalent of [Cp_2_Nb(OH)Cl_2_] or [Nb(NTA)_2_]^−^ were recorded for solutions in Hepes buffer (10 mM, pH 7.4) at 310 K. UV/Vis spectra were recorded at 5 minutes intervals against the same buffer solution containing the same amount of [Cp_2_Nb(OH)Cl_2_] or [Nb(NTA)_2_]^−^ in the reference cuvette.

 First-order rate constants, *k*_obs_, were calculated by fitting plots of absorbance at 245 nm versus time to (1) using the program Origin7.5, where *A*, *A*_0_, and  *A*_*∞*_ are the absorbances at time *t, *time zero, and after infinite time:

(1) $$\log\;\left(A_{\infty }-A\right)=-k_{\text{obs}}t+\log\;\left(A_{\infty }-A_{0}\right).$$

For titration experiments, solutions were prepared by diluting aliquots of a stock apo-Fbp solution to ~10 *μ*M with 10 mM Hepes buffer, 5 mM phosphate, pH 7.4. Aliquots of metal complex (0.5–10 *μ*l) were added, and each solution was allowed to equilibrate at 310 K for 1 hour before the spectrum was recorded. 

 The displacement of metal ions from the protein was also monitored by adding aliquots of 1 : 50 Nb : NTA to (iron-bound) holo-Fbp in the above buffer at 310 K. UV-visible spectra were recorded half an hour after each addition. Buffers containing the same amount of 1 : 50 Nb : NTA were used as references. The binding or release of Fe^III^ was monitored by the increase or decrease in absorbance at 481 nm.

### 2.5. Chromatographic Analysis

For chromatographic analysis, the Nb-Fbps were prepared by reacting apo Fbp with 20 mol equiv of freshly prepared Cp_2_NbCl_2_ (bubbled with air, as oxygen is required to effect oxidation to the water soluble niobium(V) complex Cp_2_Nb(OH)Cl_2_) or [Nb(NTA)_2_]^−^ for 48  hours at 310 K in 10 mM Hepes buffer, pH 7.4. Small molecules (<30 kDa) were removed by ultrafiltration using 0.1 M KCl, and then the sample was applied to a Mono S HR5/5 column equilibrated with Hepes buffer (10 mM, pH  7.4, 25 mL), followed by gradient elution with 0-1 M KCl in Hepes (10 mM; pH7.4) flow rate 0.5 mL min^−1^. Peak fractions were collected and pooled, and then subjected to ultrafiltration (Centricon, 30 kDa cut off, YM-30, Millipore) to remove NaCl.

### 2.6. ICP-OES Analysis

ICP-OES was performed on Perkin Elmer Optical Emission Spectrometer Optima 5300DV using standard methods. Metal-loaded proteins were prepared using the same chromatographic procedures as for holo-Fbp isolation, collected and purified by using Centricon 30 (Amicon) ultrafiltration and washing six times with ultrapure water followed by ultrafiltration after each washing. The protein solution was finally diluted with ultrapure water. The contents of Nb and S were measured, after digestion of the samples, using the emission lines of 309.418 nm and 181.975 nm for Nb and S, respectively.

### 2.7. Mass Spectrometry

Samples Nb-Fbps for ESI-MS were prepared by reacting a 20-fold molar excess of [Cp_2_Nb(OH)Cl_2_] or [Nb^V^(NTA)_2_] with apoFbp (ca. 0.5 mM) in 10 mM HEPES buffer pH 7.5 in a water bath at 310 K for 48  hours. Unbound Nb^V^ complexes were removed from the protein by ultrafiltration (Centricon 30, cut-off 30 kDa, Amicon) washing with 0.1 M KCl and H_2_O three times, respectively, and then exchanged into a 10 mM NH_4_Ac buffer (pH 8.0) by using a PD-10 column. 

 Positive-ion electrospray mass spectrometry was performed on a Micromass Platform II quadruple mass spectrometer equipped with an electrospray ion source. The purified holo-Fbp, apo-Fbp, or recombinant Nb-Fbp samples in 10 mM NH_4_Ac buffer (pH 8.0) were diluted with CH_3_CN/H_2_O (1 : 1, v/v) to a final concentration of 25 *μ*M. Each sample was infused at 50 *μ*L/minute directly into the mass spectrometer, and the ions were produced in an atmospheric pressure ionization (API)/ESI ion source. The spray voltage was 3.50 kV. The cone voltage was varied from 20 to 60 V as required. The capillary temperature was 338 K for direct infusion, with a 450 L h^−1^ flow of nitrogen drying gas. The quadrupole analyzer, operated at a background pressure of 5.9 × 10^−5^ mBar, was scanned at 200 Da s^−1^ for direct infusion. Data were collected (for 10 scans during the direct infusion assays) and analyzed on a Mass Lynx (ver.3.5). The deconvoluted average molecular mass was determined using the MaxEnt and Transform algorithms of massLynx software.

### 2.8. X-Ray Absorption Spectroscopy

X-ray spectra were recorded at the niobium K edge on EXAFS station 16.1 at Daresbury Laboratory Synchrotron Radiation Source (operating at 2 GeV) using an Si 〈220〉 double crystal monchromator and vertically focusing mirror for harmonic rejection. Data for apo-Fbp loaded with Nb^V^ were collected at 13 K (using a liquid helium cryostat) in fluorescene mode using a 13-element solid state germanium detector. Data were collected in *k* space using a *k^3^-*weighted regime for counting time with a total scan time of 40 minutes. 40 scans were collected from each sample. The edge positions were calibrated against an Nb foil. Samples were prepared as follows. Purified native Fbp was concentrated to 5 mM by ultrafiltration and washed six times with 0.1 M KCl. Nb-Fbp (1 mM) was prepared by reacting apo-Fbp with 10 mol eq of Cp_2_Nb(OH)Cl_2_ in Hepes buffer (pH 7.4). The excess of [Cp_2_Nb(OH)Cl_2_] was removed from the yellowish solution by ultrafiltration, washing three times with 0.1 M KCl and then ultrapure water. 

 Data were processed using EXCALIB and SPLINE (modified for use with EXCURV) [25]. The EXAFS data were converted into *k* space and analyzed using the fast curved wave (or Rehr-Albers) theory [26] including up to third-order multiple scattering contributions in EXCURV98 [27]. Phase shifts were calculated using Hedin-Lundquist exchange and correlation potentials [28, 29] and tested against the EXAFS data for [Cp_2_NbCl_2_] and NbCl_5_. All the data analysis was conducted on raw EXAFS data (without Fourier filtering) weighted by *k^3^* to compensate for diminishing amplitude at high *k.*

## 3. Results and Discussion

### 3.1. Characterization of Fbp

Since Fbp contains an N-terminal signal sequence that directs Fbp to the periplasmic space, the overexpressed protein was located in the periplasm of *E. coli*. The molecular masses of holo-Fbp and apo-Fbp determined by electrosprary mass spectrometry were 33,640 Da, in good agreement with the amino acid sequence (309 amino acids, without iron or phosphate theoretical mass of 33639.39 Da). This suggests that, under the conditions used for mass spectrometry, neither holo-Fbp nor apo-Fbp had iron or synergistic anion bound to the protein and that the Fbp signal sequence had been cleaved at Asp23 upon translocation to the periplasm. The presence of iron in holo-Fbp was evident from the ligand-to-metal charge-transfer (LMCT) (tyrosinate-to-Fe^III^) band of Fe^III^-Fbp (*vide infra*). For metal binding experiments, iron was efficiently removed by incubation with excess citrate to generate apo-Fbp with no detectable iron remaining.

### 3.2. Rate and Stoichiometry of Nb Binding to ApoFbp

**Figure 2:**
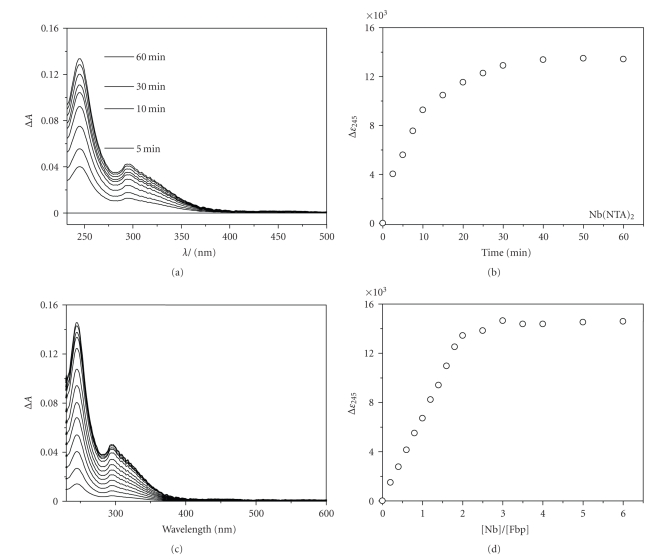
(a) UV difference UV/Vis spectra recorded at various times during the reaction of apoFbp (10 *μ*M) with 2.0 mol equivalents of [Nb(NTA)_2_]^−^ in 10 mM Hepes buffer, 5 mM phosphate, pH 7.4, 310 K. (b) Time course for reactions of apo-Fbp (ca. 10 *μ*M) with 2 mol equivalents of [Nb(NTA)_2_]^−^ in the 10 mM Hepes buffer, 5 mM phosphate, pH 7.4, 310 K, as a plot of molar absorptivity versus time for reaction. (c) Difference UV/Vis spectra for the titration of apoFbp (10 *μ*M) with [Nb(NTA)_2_]^−^ in 10 mM Hepes buffer, 5 mM phosphate, pH 7.4, 310 K (1 h equilibration). Molar ratios of Nb-complexes: apo-Fbp from bottom to top: are 0–2.0 in 0.2 mol equivalent steps, then 2.5, 3.0, 3.5, 4.0. (d) Titration curve for the reaction in (C), and Δ*ε* is the absorbance at 245 nm divided by the Fbp concentration.

**Figure 3:**
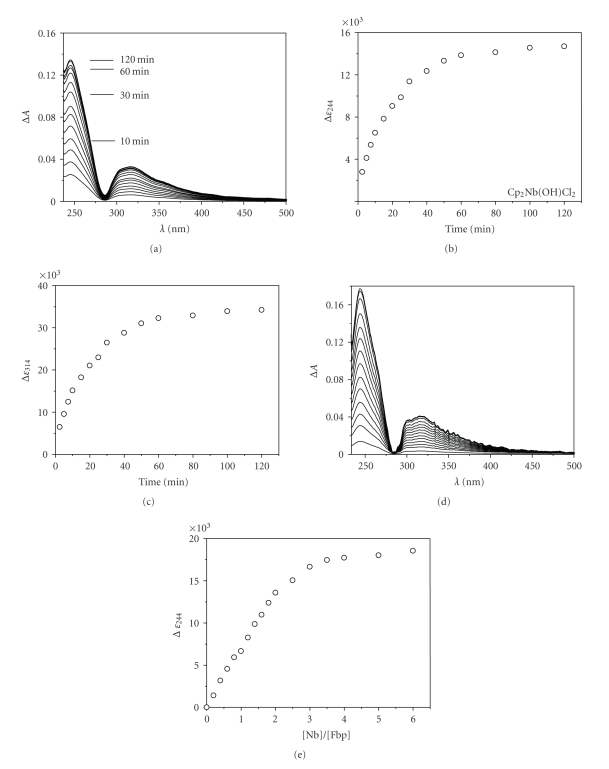
(a) Difference UV/Vis spectra recorded at various times during the reaction of apoFbp (9.8 *μ*M) with 2.0 mol equivalents of [Cp_2_Nb(OH)Cl_2_] in 10 mM Hepes buffer, 5 mM phosphate, pH 7.4 (b) Time course for reactions of apo-Fbp (ca. 9.8 *μ*M) with 2 mol equivalents of [Cp_2_Nb(OH)Cl_2_] in 10 mM Hepes buffer, 5 mM phosphate, pH 7.4, 310 K, as a plot of molar absorptivity versus time of reaction. (c) Time course for reactions of apo-Fbp (ca. 9.8 *μ*M) with 2 mol equivalents of [Cp_2_Nb(OH)Cl_2_] in the 10 mM Hepes buffer, 5 mM phosphate, pH 7.4 at 310 K as a plot of molar absorptivity versus time of reaction. (d) Difference UV/Vis spectra for the titration of apoFbp (9.8 *μ*M) with [Cp_2_Nb(OH)Cl_2_] in 10 mM Hepes buffer, 5 mM phosphate, pH 7.4, 310 K (2 h equilibration). Molar ratios of Nb-complexes: apo-Fbp from bottom to top: are 0–2.0 in 0.2 mol equivalent steps, then 2.5, 3.0, 3.5, 4.0. (e) Titration curve for the reaction in (D), Δ*ε* is the absorbance at 244 nm divided by the Fbp concentration.

The time-courses of reactions between apoFbp and [Nb^V^(NTA)_2_]^−^ (we use this formulation for solutions containing Nb^V^ and 2 mol equiv of NTA) or [Cp_2_Nb^V^(OH)Cl_2_] were studied using UV/Vis spectroscopy. Two molar equivalents of [Nb^V^(NTA)_2_]^−^ or [Cp_2_Nb^V^(OH)Cl_2_] were added to a solution of apoFbp (*∼*10 *μ*M, in 500 *μ*L 10 mM Hepes buffer, 5 mM phosphate, pH 7.4) at 310 K.; typical spectra are shown in Figures 2 and 3, respectively. In the case of [Nb^V^(NTA)_2_], the reaction produced a UV difference spectrum which is similar to reactions of other metal ions with apoFbp [20, 30]; two new positive bands appeared at ca. 245 and 295 nm, and increased in intensity over a period of 60 minutes (Figures 2(a) and 2(b)). These bands are assignable to *π*-π* transitions of Tyr residues deprotonated by binding to Nb^V^. Similar bands are seen when both bacterial and serum transferrins bind to a wide variety of metal ions [31–33]. This suggests that Nb^V^ ions can occupy specific Fe^III^ binding sites. Best fits to the data were obtained using first-order kinetics equations, although the rate law was not investigated. The first-order rate constant, *k*_obs_, was 3.03 ± 0.01 h^−1^ (310 K), and the extinction coefficient (Δ*ε*_245_) reached ca. 15200 M^−1^ cm^−1^(Figure 2(c))_._

 In the case of [Cp_2_Nb^V^(OH)Cl_2_]_,_ the reaction produced two new bands in the UV/Vis spectra; one broad band is centred at around 320 nm. The other sharp band is at ca. 244 nm (Figure 3(a)). The reaction was complete in ca. 2  hours (Figures 3(a), 3(b) and 3(c)). Kinetic studies revealed *k*_obs_ of 1.24 ± 0.03 h^−1^ (310 K), and the extinction coefficient (Δ*ε*_244_) reached ca. 16700 M^−1^ cm^−1^ (Figure 3(d)). These data indicate that the reactions occur in two kinetic phases; however, the initial phases of the reactions of apoFbp with [Nb(NTA)_2_]^−^ and [Cp_2_Nb^V^(OH)Cl_2_] were fast (within 1 hour and 2 hours, resp.). 

 Analysis of the titration curves for the reaction of apoFbp with [Nb(NTA)_2_]^−^ and [Cp_2_Nb(OH)Cl_2_] (Figures 2(d) and 3(e)) suggests that about two Nb^V^ ions bind strongly to Fbp in both cases. In these experiments, each sample was allowed to equilibrate for 2  hours after each addition and then UV difference spectra were recorded. The absorptivity Δ*ε*_244/245_ increased linearly with increase in molar ratio *r*_[Nb]/[apoFbp]_ until a value of ca. *r* = 2. Beyond *r* = 2, the titration curve reached a plateau (Figures 2(d) and 3(e)). Titration studies suggested that a niobium : protein molar ratio of 2 : 1 is sufficient to deprotonate both Tyr 195 and Tyr 196 when phosphate is present as the synergistic anion; phosphate is known to bind in the interdomain cleft of the apo-protein and may prepare the cleft for metal entry [34]. Beyond a 2 : 1 [Nb] : [apoFbp] ratio there was little increase in the absorption at 244 or 245 nm (Figures 2(d) and 3(e)). These data suggest that the initial reaction with apo-Fbp involves Nb^V^ binding to Tyr 195 and Tyr 196 either initially as a mononuclear niobium centre binding to one of the Tyr side-chains followed by subsequent binding of the second niobium to the second Tyr and formation of an oxo-niobium dinuclear center or perhaps by direct uptake of a dinuclear species which may involve one Nb^V^ binding to both Tyr residues or one to each. Hence there is little increase in the absorption at 241 nm beyond a 2 : 1 [Nb] : [apoFbp] ratio. In particular, the mobility of Tyr 196 (as observed in crystals of oxo-Fe^III^-Fbp) [22] may be important for capturing Nb^V^ ions at the protein surface and delivering them into the binding cleft. In our previous studies of the binding of trinuclear Fe^III^, Hf^IV^ (also pentanuclear Hf^IV^), and Zr^IV^ oxo-clusters to Fbp, we have observed anchoring of these centres via these tyrosinates with each binding to different metals (or in the case of one Fe^III^ cluster, anchoring via a single Tyr) [20, 22, 35]. It appears that the other two protein ligands, His 9 and Glu 57 are not essential for the initial steps of metal binding in vitro.

### 3.3. Displacement of *F* *e* ^*III*^ from Fe-Fbp by *N* *b* ^*V*^

**Figure 4:**
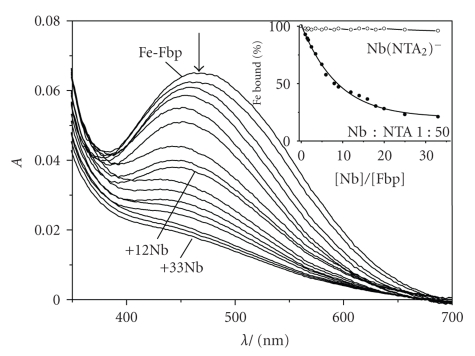
Displacement of Fe^III^ from holoFbp (25 *μ*M) by 1 : 50 Nb : NTA under the same conditions as Figure 2(a). Molar ratio of Nb/Fbp (from top to bottom) is 0, 2, 4, 6, 8, 10, 15, 20, 25, 30, and 33. insert shows the percentage of Fe^III^ bound to the protein calculated from the LMCT band at 465 nm. Little Fe^III^ is displaced by [Nb(NTA)_2_]^−^ (open circles) whereas 1 : 50 Nb : NTA gives rise to almost complete displacement of Fe^III^ from holoFbp (filled circles).

To confirm that Nb^V^ binds to the specific Fe^III^-binding sites of Fbp/Ng, we investigated the displacement of Fe^III^ from holo-Fbp (Fe^III^-phosphate-Fbp) by Nb^V^. Holo-Fbp was saturated with Fe^3+^ by incubating apoFbp (100 *μ*M) with 10 mol equivalent 1 : 2 Fe : NTA in 10 mM Hepes, 5 mM phosphate, pH 7.4, 310 K for 24  hours. After removing unbound Fe and diluting the Fe-Fbp solution to 25 *μ*M, 1.0 mol equivalent of [Nb(NTA)_2_]^−^ was added to the holo-Fbp solution in physiological buffer at 310 K. There was no obvious change to the LMCT band of Fe^III^-Fbp at 465 nm (Figure 4, insert). In contrast, with the addition of 1 : 50 Nb : NTA, this band decreased in intensity to about half of its original value at the mol ratio of *r*_[Nb]/[Fe-Fbp]_ of 10, and to about 21% when *r*_[Nb]/[Fe-Fbp]_ is 33 (Figure 4). It can be seen from the graph that the wavelength of the absorption maximum of the LMCT band shifted from 480 nm to 465 nm during this titration. This suggests that under these conditions phosphate is displaced form iron [30] and that Nb^V^ can compete with Fe^III^ for binding to Fbp, perhaps with initial formation of mixed-metal oxo-Fe/Nb species in the binding cleft.

### 3.4. Displacement of *N* *b* ^*V*^ from Nb_2_-Fbp by *F* *e* ^*III*^

**Figure 5:**
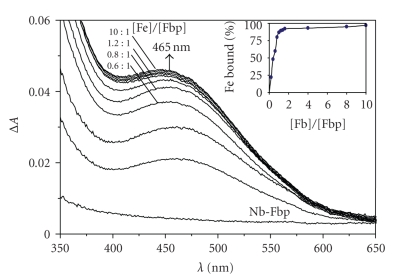
Displacement of Nb^V^ from Nb_2_-Fbp (prepared by incubating apoFbp (100 *μ*M) with 10 mol equiv 1 : 2 Nb : NTA under the same conditions as Figure 2(a)) by 1 :  2 Fe : NTA in the same buffer at 310 K. the band at ca.465 nm increases in intensity with the addition of Fe^III^. Molar ratios *r*_[Fe]/[Fbp]_ from bottom to top: are 0, 0.2, 0.4, 0.6, 0.8, 1.0, 1.2, 1.4, 1.6, 4, 8, and 10. Insert shows variation in percentage of Fe^III^ bound to the protein calculated from the LMCT band at 465 nm with the amount of added Fe^III^.

We also investigated the displacement of Nb^V^ from Nb-Fbp by Fe^III^. Nb-Fbp was prepared by incubating apo-Fbp (100 *μ*M) with 10 mol equiv equivalent 1 : 2 Nb : NTA in 10 mM Hepes, 5 mM phosphate^−^, pH 7.4, 310 K for 24  hours, and Fe^III^-binding was monitored by the appearance of the Fe^III^-Fbp LMCT band at 465–480 nm. Nb-Fbp was titrated with 0.2–10 mol equiv 1 :  2 Fe : NTA in the same buffer solution at the same temperature. UV difference spectra were recorded 0.5  hours after each addition of Fe^III^. A peak centred at ca. 465 nm appeared and increased after 1.2 mol equiv of 1 :  2 Fe : NTA had been added, at which point it had almost reached its final intensity and no further increase occurred with 10 mol equiv Fe^III^ present (Figure 5). Therefore, under the conditions studied here, Fe^III^ can displace Nb^V^ from the protein.

### 3.5. Characterization of Nb-Fbp by Ion Exchange Chromatography and ICP-OES Analysis

**Figure 6:**
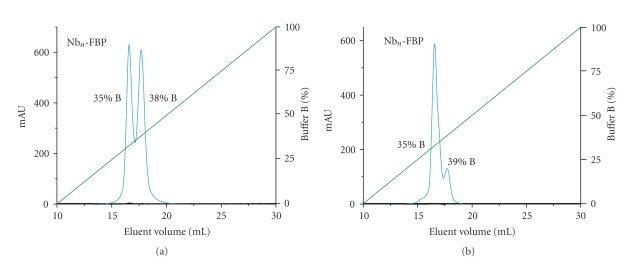
Characterization of Nb_n_-FBP (apo-Fbp reloaded with [Cp_2_Nb(OH)Cl_2_] (a) or [Nb(NTA)_2_] (b)) by chromatography on a MonoS HR 5/5 column. The left axis shows the absorption profile at 280 nm, and the right axis is the percentage of buffer B (10 mM Hepes, 1 M NaCl, pH7.5). Green line: gradient of the buffer B applied during the elution process; cyan line: absorbance at 280 nm.

The products from reaction of apoFbp with 20 molar equiv of freshly prepared [Cp_2_NbCl_2_] (bubbled with air (oxygen) to obtain the water soluble niobium(V) complex [Cp_2_Nb(OH)Cl_2_]) or [Nb(NTA)_2_]^−^ are at 310 K in Hepes buffer (10 mM, pH 7.4) for 48  hours. Unbound Nb was removed by extensive ultrafiltration and the sample was applied to a cation exchange Mono S HR5/5 column equilibrated with Hepes (10 mM; pH 7.4, 25 mL), followed by gradient elution with 0-1 M KCl in Hepes (10 mM; pH 7.4) flow rate 0.5 mL min^−1^. The chromatograms are shown in Figure 6. We observed two peaks that elute at different KCl concentrations which we assume to be the different forms of Nb-Fbp with different charges. It seems likely that the products from these loading reactions contain different multinuclear forms of Nb-Fbp. 

 Reaction of Fbp with 1 and 20 mol equivalents of [Cp_2_Nb(OH)Cl_2_] gave products containing an average of 0.86 and 2.23 mol Nb per mol protein, respectively (ICP-OES data; see Table 1). In the case of reactions with 1 and 20 mol equivalents of [Nb(NTA)_2_]^−^ under the same conditions, the products contained ca. 0.91 and 2.41 mol Nb per mol protein.

### 3.6. Electrospray Ionization Mass Spectroscopy (ESI-MS)

The recombinant protein, prepared as described in experimental section, was further studied by electrospray ionization mass spectrometry. The products were investigated as dilute solutions in 10 mM NH_4_Ac, pH 8.0. For the reaction of [Cp_2_Nb(OH)Cl_2_] with apoFbp, peaks centred at mass 34813, 34829, 34842, 34871, and 34890 are tentatively assigned to [aFbp + 4Cp_2_NbCl_2_], [aFbp + 4Cp_2_NbCl_2 _+ OH^−^], [aFbp + 4Cp_2_NbCl_2_ + 2OH^−^], [aFbp + 4Cp_2_NbCl_2_ + 3OH^−^], and [aFbp + 4Cp_2_Nb(OH)Cl_2_], respectively. Another sample prepared from apoFbp and [Nb(NTA)_2_]^−^ gave peaks centred at mass 34655, 34837, and 34965 corresponding to [aFbp + NH_4_^+^], [aFbp + 2Nb(NTA)_2_ + CH_3_COO^−^], [aFbp + 3Nb(NTA)_2_ + NTA]. Table 2 contains a list of the species observed by ESI-MS assays of Nb-Fbp. These results indicate that apoFbp binds Nb^V^ tightly under the conditions used and suggests that the binding cleft can accommodate not only a single metal ion but also multinuclear Nb species, as observed previously for iron, zirconium and hafnium. However, these data alone do not rule out the possible presence of Nb binding sites elsewhere on the protein and the formulations require further verification before they can be fully interpreted.

### 3.7. EXAFS Experiment

**Figure 7:**
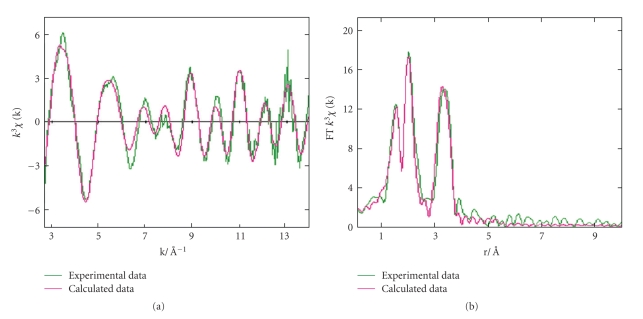
Nb K-edge X-ray absorption data. (a) EXAFS spectra, and (b) Fourier transform data for Nb-Fbp (prepared from 10 : 1 [Cp_2_Nb(OH)Cl_2_]  : apoFbp reaction). *Green line*: experimental data; *red line: *calculated data.

To obtain more detailed structural information, Nb K-edge X-ray absorption near-edge structure (XANES) and extended X-ray absorption fine structure (EXAFS) studies (Figure 7) were carried out. For these studies, Nb-Fbp was prepared by treating apo-Fbp with [Cp_2_Nb(OH)Cl_2_] (see experimental section). 

 The XANES edge position confirms the oxidation state of the Nb as 5+. The Fourier transform shows two intense, overlapping peaks at ca. 1.94 Å and 2.12 Å, and a broader peak at 3.3 Å. The 1.94 Å and 2.12 Å peaks were simulated with six oxygen atoms as back-scatters at two distances (2 atoms at 1.94 Å, and 4 atoms at 2.12 Å). These result from scattering from the atoms directly coordinated to the Nb^V^ center and are likely to include tyrosinate, histidine, glutamate, hydroxide, and oxo groups. ICP measurements showed that phosphate was not present in this sample (data not shown). Niobium-aryloxide bonds of 1.730–1.985 Å have previously been reported, including Nb-O_phenolate_ distances of ca. 1.872 Å [36]. Thus the two shorter inner-sphere Nb-O bonds in Nb-Fbp (1.94 Å) are within the range of typical Nb-O bonds assignable to the Nb-tyrosinate bonds but may also be due to Nb=O double bonds and a possible bridging oxo group. The Nb-O bond lengths are also similar to those in known compounds such as (C_2_H_6_NO_2_)_2_[NbOF_5_] and (C_3_H_8_-NO_2_)_2_[NbOF_5_]·2H_2_O [37], LiNbO(O-2,6-PhMe_2_)_4_P·3THF, LiNbCl_3_(O-2,6-PhMe_2_)_2_P·2THF [38], NbCl_3_(3-[2,2′-methylenebis(4,6-di-*tert*-butylphenol)-5-*tert*-butylsalicylidene-(2,6-diisopropyl)phenylimine]) [34] and the [(Nb_6_Cl_8_O_4_)Cl_6_] cluster. The Nb-Nb peak at 3.3 Å is similar to that reported for the single Nb neighbor at 3.3 Å in a niobium-peroxo-citrato complex [39]. The EXAFS data may be consistent with the presence of a dinuclear Nb-O-Nb centre in the adduct. No attempt was made to include Cp ligands in a fit to the EXAFS data. 

 The EXAFS data do not allow an unambiguous assignment of a structure to the bound niobium(V) dinuclear centre since only averaged Nb–N/O bond lengths are obtained and oxygen ligands cannot be distinguished from nitrogen donors. One possibility is that the dinuclear centre is anchored to the protein only by coordination to the two active site Tyr residues, as are the clusters in previously characterised Fe, Zr, and Hf complexes. The other ligands for Nb^V^ may be oxygens from water or hydroxide without coordination to the His or Glu sidechains which are ligands in the mononuclear Fe^III^ site.

## 4. Conclusions

Previous work has shown that efficient iron acquisition is required for the virulence of pathogenic bacteria and that Fbp is one of the iron-uptake virulence genes [22, 30, 35, 40–43]. The di-tyrosyl metal-binding motif in Fbp is highly conserved and shows a strong binding ability with some metals. Targetting this protein with an unnatural metal ion such as niobium(V) which might block iron(III) uptake therefore becomes a potential strategy for the design of novel antibiotics. 

 Since the size of the binding cleft in Fbp is thought to be matched to Fe^III^ (ionic radius 0.69 Å), previous studies have shown similar tight binding between apoFbp and various other metal ions such as Ti^IV^ (ionic radius 0.75 Å), Zr^IV^ (ionic radius 0.86 Å) and Hf^IV^ (ionic radius 0.85 Å). Hence it seemed reasonable to suppose that Nb^V^ (ionic radius 0.78 Å) might behave in a similar manner to these other metal ions. As we expected, Nb^V^ from the [Nb(NTA)_2_]^−^ and antitumor complex [Cp_2_NbCl_2_]/[Cp_2_Nb(OH)Cl_2_] were readily taken up into the specific iron sites of ferric iron binding protein. The tight binding was confirmed by ICP-OES and ESI-MS studies. Interestingly, kinetic studies showed that the uptake of Nb^V^ by apo-Fbp is relatively rapid in vitro under the conditions used, and titration studies monitored using UV/vis also show that Nb^V^ can be displaced by Fe^III^, although suggesting weaker binding of Nb^V^. Structural studies using EXAFS suggest the presence of a dinuclear Nb(V) centre possibly with an Nb-O-Nb bridge, but further studies are needed to define the conditions under which the Cp ligands are displaced from Nb on binding to the protein. These properties may allow Nb^V^ to bind strongly to Fbp under certain environmental conditions. It would also be interesting to investigate potential catalytic properties of such a protein-bound dinuclear niobium centre. The recent novel use of the metal complex desferrioxamine-gallium (DFO-Ga) that targets *P. aeruginosa* iron metabolism and stops biofilm formation [44], coupled with our work, suggests that Nb^V^ complexes should be explored as potential novel metalloantibiotics.

## Figures and Tables

**Table 1:** Analysis of the products from reactions of apo-Fbp with [Cp_2_Nb(OH)Cl_2_] and [Nb(NTA)_2_]^−^.

Reaction mixture^(a)^	Product^(b)^	Reaction mixture	Product
[Cp_2_Nb(OH)Cl_2_]/[Fbp]	[Nb]/[Fbp]	[Nb(NTA)_2_]^−^/[Fbp]	[Nb]/[Fbp]
1 : 1	0.86 ± 0.10 : 1	1 : 1	0.91 ± 0.10 : 1
20 : 1	2.23 ± 0.10 : 1	20 : 1	2.41 ± 0.10 : 1

^(a)^ Reactions carried out for 48  hours in 10 mM HEPES buffer, pH 7.4, 310 K.

^(a)^Fbp concentration determined from A_280_.

**Table 2:** Species detected by ESI-MS assay of products from the reaction of [Nb(NTA)_2_]^−^ or [Cp_2_Nb(OH)Cl_2_] with apo-Fbp.

Calcd/Da^(a)^		Obsd/Da
Recombinant apo-Fbp with [Cp_2_Nb(OH)Cl_2_]		
[a-Fbp]	(33640)	33648
[aFbp + 4Cp_2_NbCl_2_]	(34816)	34813
[aFbp + 4Cp_2_NbCl_2_+OH^−^]	(34833)	34829
[aFbp + 4Cp_2_NbCl_2_+2OH^−^]	(34850)	34842
[aFbp + 4Cp_2_NbCl_2_+3OH^−^]	(33867)	34871
[aFbp + 4Cp_2_Nb(OH)Cl_2_]	(34884)	34890

Recombinant apo-Fbp with [Nb(NTA)_2_]^−^		

[aFbp + NH_4_^+^]	(33657)	33655
[aFbp + 2Nb(NTA)_2_ + NTA + CH_3_COO^−^]	(34835)	34837
[aFbp + 3Nb(NTA)_2_ + NTA]	(34966)	34965

^(a)^The formulations are merely those which give reasonable fits to the observed masses and cannot be interpreted as giving structural information about the nature of the bound complexes. For example some sites could be on the exposed surface of the protein as well as in the interdomain cleft.
